# LncRNA ENSMUST_147219 mediates the progression of ischemic acute kidney injury by targeting the miR-221-5p/IRF6 axis

**DOI:** 10.1007/s10495-022-01730-3

**Published:** 2022-05-26

**Authors:** Jing Liu, Xiaozhou Li, Jurong Yang, Dongshan Zhang

**Affiliations:** 1 Department of Nephrology, The Third Affiliated Hospital of Chongqing Medical University, Chongqing, 401120 People’s Republic of China; 2 Department of Emergency Medicine, Second Xiangya Hospital, Central South University, Changsha, Hunan People’s Republic of China; 3 Emergency Medicine and Difficult Diseases Institute, Second Xiangya Hospital, Central South University, Changsha, Hunan People’s Republic of China; 4 Department of Nephrology, Second Xiangya Hospital, Central South University, Changsha, 410011 Hunan People’s Republic of China

**Keywords:** AKI, LncRNA, miR-221-5p, IRF6, Apoptosis

## Abstract

Although previous studies have revealed that long noncoding RNAs (lncRNAs) regulate the progression of ischemic acute kidney injury (AKI), the exact role and mechanism of lncRNA ENSMUST_147219 in ischemic AKI are not clear. In the present study, lncRNA ENSMUST_147219 was induced by ischemic injury in vitro and in vivo. Functionally, lncRNA ENSMUST_147219 mediated apoptosis in mouse proximal tubule‐derived cell line (BUMPT). Mechanistically, lncRNA ENSMUST_147219 sponged the microRNA (miR)-221-5p to upregulate the expression of interferon regulatory factor 6 (IRF6) to drive apoptosis. Finally, knockdown of lncRNA ENSMUST_147219 markedly attenuated the ischemic AKI by targeting the miR-221-5p/IRF6 axis. Collectively, our data demonstrated that lncRNA ENSMUST_147219 promoted the development of ischemic AKI by regulating the miR-221-5p/IRF6 pathway, which could be considered a new therapeutic target for ischemic AKI.

**Supplementary Information:**

The online version contains supplementary material available at 10.1007/s10495-022-01730-3.

## Introduction

Acute kidney injury (AKI), an abrupt decrease in kidney function, affects about 7% of all hospitalized patients and more than 35% of intensive care patients [1]. The mortality of AKI in critically ill patients is more than 50% [2]. Ischemia/reperfusion (I/R), sepsis, and nephrotoxicity are usually considered the primary causes of AKI [3–5]. Among them, ischemic injury is the main cause of AKI. Although the pathophysiology of ischemic AKI remains largely unknown, it has been reported that renal cell apoptosis contributes to the progression of ischemic AKI [6]. Most studies have found that several key proteins, such as p53, MBD2, PKCδ, DsbA-L, and Wnt, mediate renal cell apoptosis [4, 5, 7–11]. However, the mechanism of renal cell apoptosis is not completely understood.

Long noncoding RNAs (lncRNAs) are more than 200 nucleotides in length [12, 13]. They usually serve as competing endogenous RNAs (ceRNAs) to regulate the activity of microRNAs (miRNAs) [14]. miRNAs, especially 22-nt endogenous RNAs, target mRNAs to suppress translational activity [15]. Emerging evidence shows that lncRNAs participate in the pathophysiological regulation of ischemic AKI. One study demonstrated that lncRNA TUG1 suppressed renal tubular cell apoptosis [16]. Other studies showed that lncRNA MEG3, lnc NEAT1, lnc GAS5, and lncRNA H19 promoted renal tubular cell apoptosis [17–20]. However, the role and regulatory mechanism of lncRNAs in ischemic AKI remain largely unknown.

In the present study, we reported that lncRNA ENSMUST_147219 mediated I/R-induced renal tubular cell apoptosis. Mechanistically, lncRNA ENSMUST_147219 acted as a ceRNA to sponge miR-221-5p and then increase the expression of interferon regulatory factor 6 (IRF6). Finally, knockdown of lncRNA ENSMUST_147219 attenuated I/R-induced AKI by regulating the miR-221-5p/IRF6 pathway.

## Materials and methods

### Antibodies and reagents

IRF6 (MA5-38051) was obtained from Thermo Fisher Scientific (Waltham, MA, USA). β-Tubulin (Cat. No. AF7011) was purchased from Affinity Bioscience (Rosemont, IL, USA). Caspase-3 (Cat. No. 9662S) and cleaved caspase-3 (Cat. No. 9664) were purchased from Cell Signaling Technology (Danvers, MA, USA). The luciferase assay kit was purchased from BioVision (Milpitas, CA, USA). The fluorescein isothiocyanate (FITC) Annexin V Apoptosis Detection Kit I (Cat. No. 556547) was obtained from BD Pharmingen (Franklin, NJ, USA).

### Cell culture and treatments

BUMPT cells were cultured in DMEM (Gibco) supplemented with 10% fetal bovine serum (FBS) and 1% penicillin–streptomycin (10,000 U/mL and 10,000 g/mL, respectively), and incubated at 37 °C in a humidified atmosphere of 5% CO_2_. BUMPT cells were treated with 10 μM antimycin A and 1.5 μM calcium ion carrier in Hanks’ balanced salt solution (Hyclone, SH30030.02) for 2 h followed by the replacement with complete DMEM medium for 0, 2 h, and 4 h in the reperfusion stage. In addition, BUMPT cells were transfected with miR-221-5p antagomir (100 nM), miR-221-5p mimic (100 nM), ENSMUST_147219 siRNA (100 nM), IRF6 siRNA (100 nM), or negative control (Ruibo, Guangzhou, China) using Lipofectamine 2000 (Life Technologies, Carlsbad, CA, USA).

### Luciferase reporter assays

The luciferase reporter assays were performed as previously described [21]. The luciferase vectors for lncRNA ENSMUST_147219 (WT-Luc-ENSMUST_147219) and IRF6-3’UTR (WT-Luc-IRF6), but not the mutated plasmids for ENSMUST_147219 (MUT-Luc-ENSMUST_147219) and IRF6 (MUT-Luc-IRF6), contained the complementary sequence of miR-221-5p. Renilla luciferase (RLuc) was used as an internal control. All plasmids were constructed by RuQi Biotechnology (Guangzhou, Guangdong, China). The pGMLR-TK plasmid with WT-Luc-ENSMUST_147219 or MUT-Luc-ENSMUST_147219 with or without miR-221-5p mimics were co-transfected into BUMPT cells for 48 h, and then the luciferase reporter assay was conducted as previously described [22–24]. SpectraMax M5 (Molecular Devices, Sunnyvale, CA, USA) was applied to assess the gene reporter activity normalized to the RLuc signal.

### Animal model

The bilateral renal arteries of C57BL/6J mice (male, aged 8–10 weeks) were clamped for 30 min at a 37 °C heated operation table, followed by reperfusion for 24 h, 48 h and 72h. For the gene intervention experiment, C57BL/6J mice were preinjected with lncRNA ENSMUST_147219 siRNA or control siRNA via the tail vein for 12 h before ischemic injury. The saline injection or sham operation was considered the control group. The blood samples and renal tissues were collected for the analyses of renal function and morphology. The animal experiments were carried out in accordance with the recommendations of the Institutional Committee for the Care and Use of Laboratory Animals of Second Xiangya Hospital (China). All of the animals were provided access to food and water at any time and were housed on a 12-h light/dark cycle.

### Renal function, morphological studies, and apoptosis

The assessment of blood urea nitrogen (BUN) and serum creatinine was performed in accordance with the manufacturer’s procedure (Nanjing Jiancheng Bioengineering Institute, Jiangsu, China). H&E staining was applied to assess the degree of renal tissue damage, the criteria of tubular damage contained brush border, the loss of tubular dilation, cast formation, and cell lysis [24, 25]. The percentage of damaged tubules was classified as follows: 0, no damage; 1, < 25% damage; 2, 25–50% damage; 3, 51–75% damage; 4, > 75% damage. Renal cell apoptosis was assessed by TUNEL staining and then quantified by calculating the percentage of positively stained cells in accordance with a previous study [25]. An Olympus microscope (Olympus Corporation, JPN) equipped with UV epi-illumination was used to analyze the stained samples. Flow cytometry (FCM) procedures were performed in accordance with the manufacturer’s protocol.

### Relative quantification PCR

Total RNA from BUMPT cells and kidneys of C57BL/6J mice was extracted using a Trizol reagent (Accurate Biology, ChangSha, China) kit [8, 26, 27]. Total RNA (40 ng) was reverse-transcribed using Moloney Murine Leukemia Virus (M-MLV) Reverse Transcriptase (Accurate Biology). Real-time qPCR was used to examine the expression levels of miRNA, mRNA, and lncRNA by Bio-Rad (Hercules, CA, USA) iQ SYBR Green Supermix with Opticon (Accurate Biology, ChangSha, China) per the manufacturer’s instructions. The sequences of lncRNA ENSMUST_147219 were obtained from Ensembl database and those of miR-221-5p were obtained from miRDB. We used the following primers: ENSMUST_147219: 5′-TTTCACCCGCTTT GCCAAGTCTC-3′ (forward) and 5′-ATCCCTTCCCTGTCTCCTCAACTG-3′ (reverse); miR-221-5p: 5′-GCGACCTGGCATACAATGTAGAT-3′ (forward) and 5′-AGTGCAGGGTCC GAGGTATT-3′ (reverse); IRF6: 5′-ACAAACTGCTCTTCTATGGGCTTCTG-3′ (forward) and 5′-TCCTCCTCCTCATCTTCATCCACATC-3′ (reverse); β-actin: 5′-GGCTGTATTCCCCTCCATCG-3′ (forward) and 5′-CCAGTTGGTAACAATGCCATGT-3′ (reverse); and U6: 5′-CTCGCTTCGGCAGCACA-3′ (forward) and 5′-AACGCTTCACGAATTTGCGT-3′ (reverse). ΔCt values were used to carry out the relative quantification.

### Immunoblot analysis

Equal amounts of lysate proteins were separated by SDS-PAGE and then transferred to a PVDF membrane (Amersham, Buckinghamshire, UK) [26, 28]. The membrane was incubated with primary antibodies against IRF6, caspase-3, cleaved caspase-3, and β-tubulin, followed by incubation with secondary antibody and detection reagents. β-Tubulin acted as an internal loading control.

### FISH

The fluorescence probes of ENSMUST_147219 and miR-221-5p were synthesized by Ruibo (Guangzhou, China). For the staining, 4′,6-diamidino-2-phenylindole (DAPI) was used to stain the nuclei; U6 was used for nuclear positive control; 18S rRNA was used for cytoplasm control; ENSMUST_147219 was labeled by CY3. Briefly, the slides of BUMPT cells and mice kidneys were hybridized with probes overnight and then stained with DAPI. A laser-scanning confocal microscope was used to analyze fluorescence images.

### Statistical analyses

Quantitative data were expressed as mean ± standard deviation (SD). Comparisons of two groups were based on two-tailed Student’s *t* tests. Multiple-group comparison was done with one-way ANOVA. All statistical analyses were carried out in the SPSS package (SPSS) and GraphPad Prism software (GraphPad Prism Software). *P* < 0.05 was considered statistically significant.

## Results

### LncRNA ENSMUST_147219 is induced by ischemic injury in vitro and in vivo

**Fig. 1:**
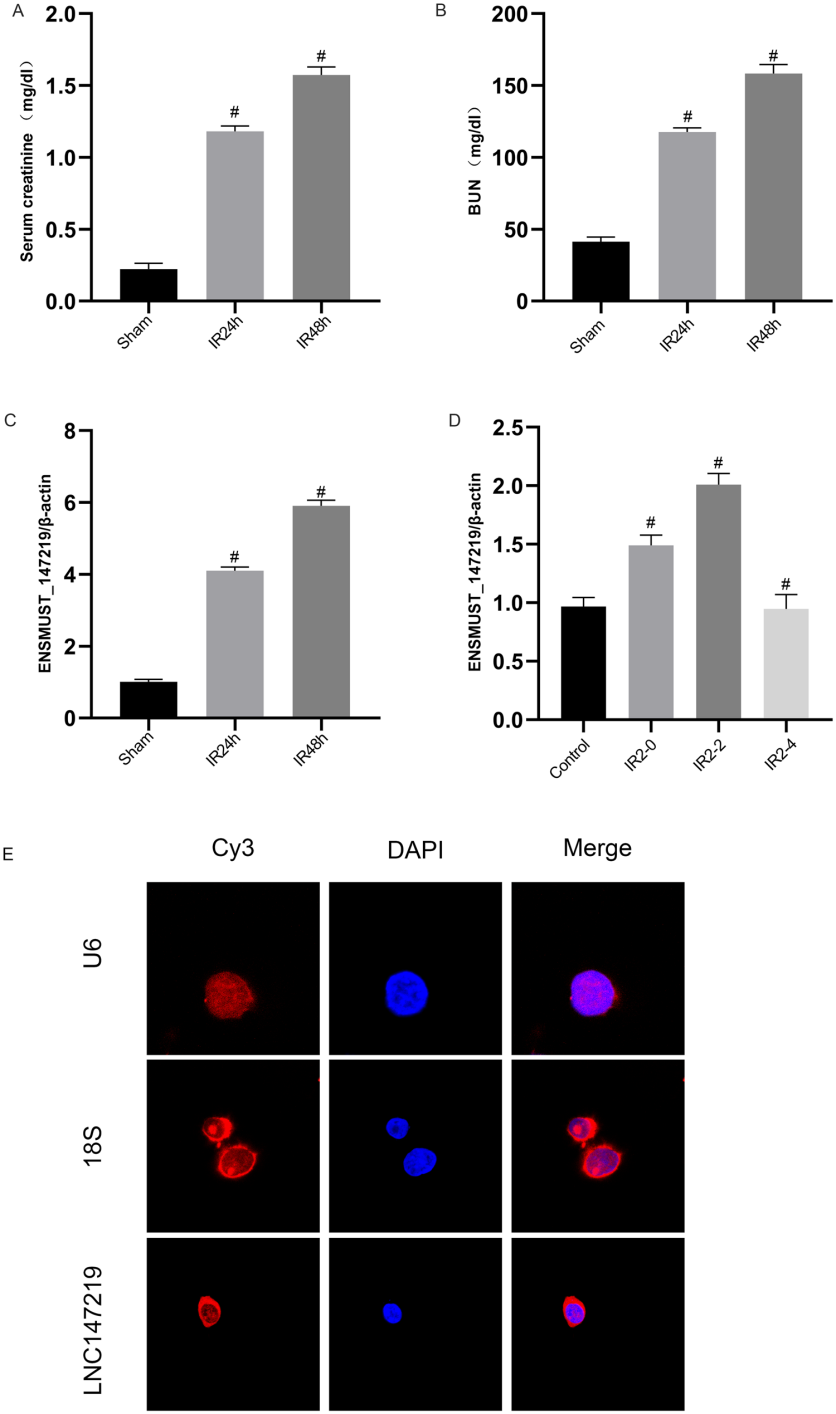
The lncRNA ENSMUST_147219 was induced by I/R in vitro and in vivo. C57BL/6J mice were subjected to I(30 min)/R(24 h, 48 h) treatment. BUMPT cells were subjected to I(2 h)/R(0, 2 h, 4 h). Blood samples were collected for the measurement of serum creatinine (**A**) and serum nitrogen (BUN) (**B**) concentrations at 24 h and 48 h. **C** and **D** RT-qPCR analysis of the expression levels of lncRNA ENSMUST_147219. **E** RNA-FISH detection of intracellular localization of ENSMUST_147219 in BUMPT cells. Data are expressed as mean ± SD (n = 6). ^#^*P* < 0.05, versus sham group or control group

We explored whether I/R could induce the expression of lncRNA ENSMUST_147219. C57BL/6 mice were subjected to ischemia for 30 min, followed by reperfusion for 24 h, 48 h and 72 h. The levels of BUN and creatinine were notably higher at 24 h after reperfusion, they further increased at 48 h (Fig. 1A, B) and declined at 72 h (Fig. S1A, B) after reperfusion. RT-qPCR analysis indicated that lncRNA ENSMUST_147219 was induced by I/R at 24 h after reperfusion and that it reached a peak at 48 h and decreased at 72 h after reperfusion (Fig. S1C). Moreover, BUMPT cells were treated with calcium and antimycin A for 2 h, followed by the reperfusion for 0, 2 h and 4 h. The RT-qPCR results indicated that lncRNA ENSMUST_147219 was slightly induced at 0 h after reperfusion, attained a peak at 2 h after reperfusion, and declined at 4 h after reperfusion (Fig. 1D). Finally, the fluorescent in situ hybridization (FISH) analysis indicated that ENSMUST_147219 was located in the cytoplasm of BUMPT cells, where U6 was used as a nuclear control and 18S was used as a cytoplasm control (Fig. 1E).

### I/R-induced renal cell apoptosis is attenuated by the knockdown of lncRNA ENSMUST_147219

**Fig. 2:**
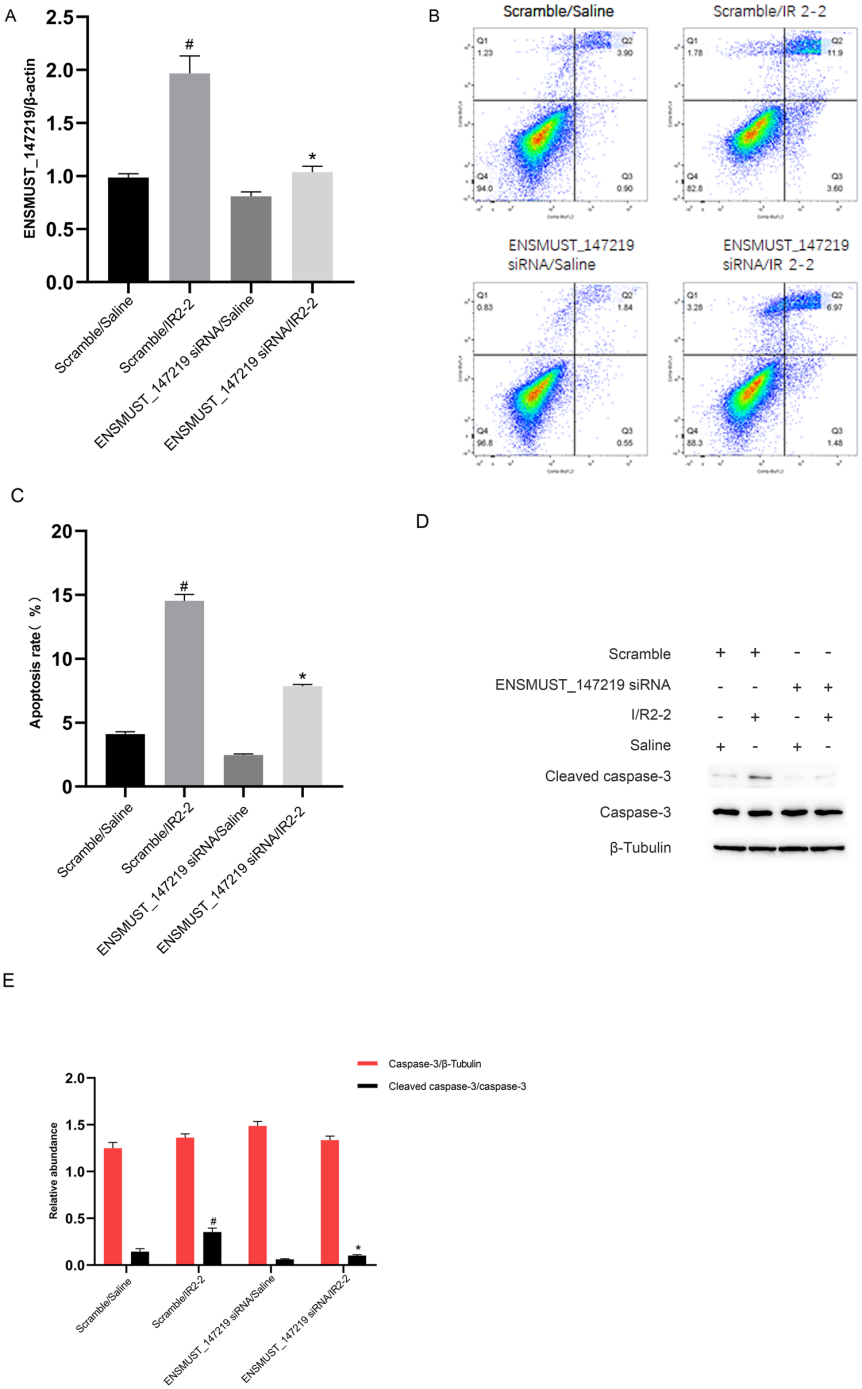
I/R-induced BUMPT cell apoptosis was ameliorated by lncRNA ENSMUST_147219 siRNA. BUMPT cells were transfected with 100 nM lncRNA ENSMUST_147219 siRNA or scramble and then treated with or without I(2 h)/R(2 h). **A** RT-qPCR analysis of the expression levels of ENSMUST_147219. **B** and **C** The FCM analysis of BUMPT cells apoptosis. **D** Immunoblot blot analysis of cleaved caspase-3 and caspase-3. **E** Densitometric measurement of immunoblot blot bands. Data are expressed as mean ± SD (n = 6). ^#^*P* < 0.05, versus scramble with saline group; *P < 0.05, versus scramble with I/R group

Next, we investigated the role of lncRNA ENSMUST_147219. LncRNA ENSMUST_147219 siRNA was transfected into BUMPT cells and then subjected to I(2 h)/R(2 h) treatment. The RT-qPCR analysis showed that lncRNA ENSMUST_147219 siRNA suppressed the expression of lncRNA ENSMUST_147219 under basic and I/R treatment (Fig. 2A). The flow cytometry (FCM) results indicated that lncRNA ENSMUST_147219 knockdown markedly reduced the I/R-induced apoptosis of BUMPT cells (Fig. 2B, C). The immunoblot analysis demonstrated that the I/R-induced increase in cleaved caspase-3 was notably reduced by the lncRNA ENSMUST_147219 knockdown (Fig. 2D, E). These data suggest that lncRNA ENSMUST_147219 is an inducer of apoptosis during ischemic injury.

### I/R-induced renal cell apoptosis is aggravated by overexpression of lncRNA ENSMUST_147219

**Fig. 3:**
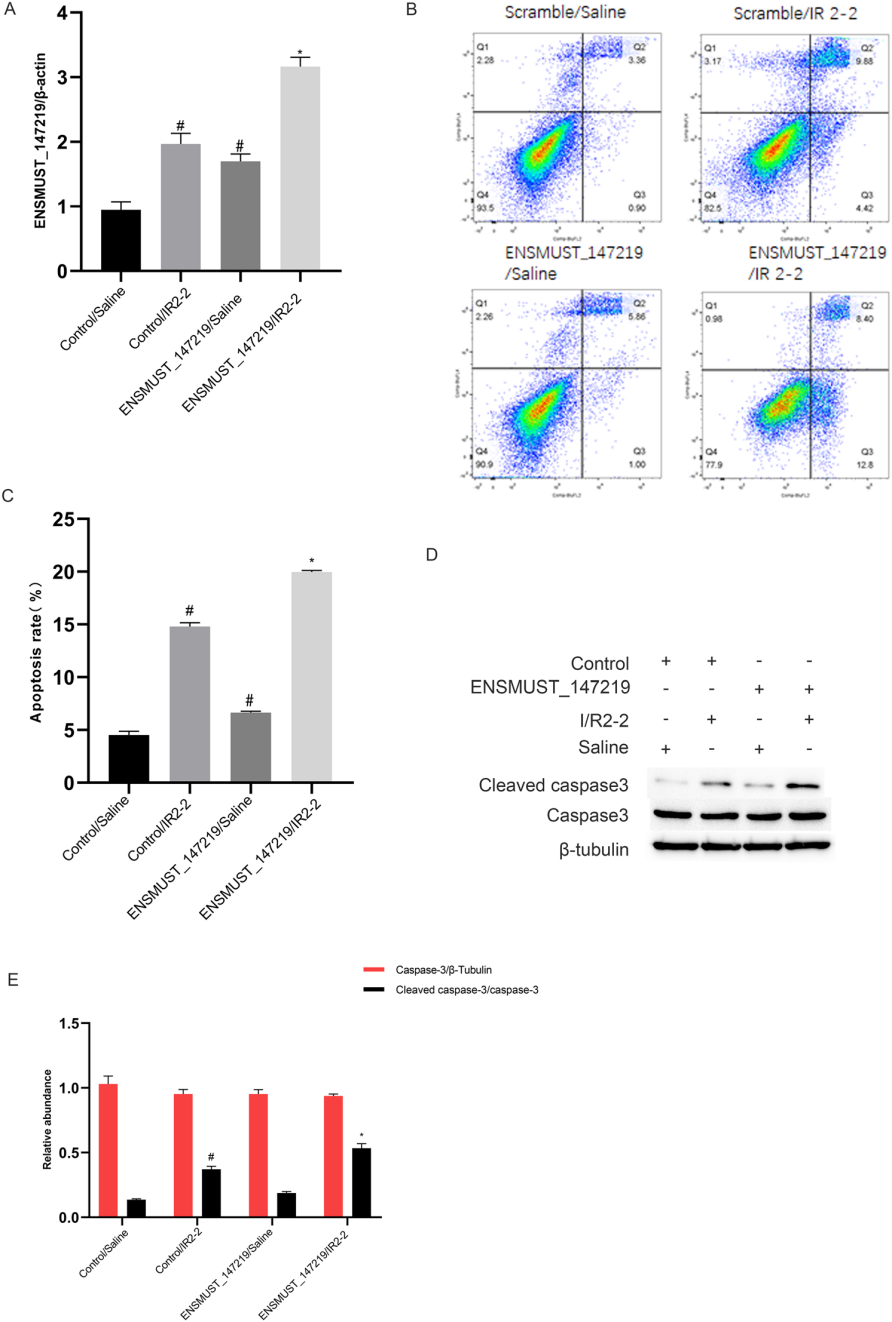
I/R-induced BUMPT cell apoptosis was reinforced by overexpression of lncRNA ENSMUST_147219. **A** BUMPT cells were transfected with lncRNA ENSMUST_147219 plasmid or control and then treated with or without I(2 h)/R(2 h). RT-qPCR analysis of the expression levels of ENSMUST_147219. **B** and **C** The FCM analysis of BUMPT cells apoptosis. **D** Immunoblot blot analysis of cleaved caspase-3 and caspase-3. **E** Densitometric measurement of immunoblot blot bands. Data are expressed as mean ± SD (n = 6). ^#^*P* < 0.05, versus scramble with saline group; *P < 0.05, versus scramble with I/R group

To further verify the proapoptotic role of lncRNA ENSMUST_147219, lncRNA ENSMUST_147219 plasmids were transfected into BUMPT cells and then subjected to I(2 h)/R(2 h) treatment. The RT-qPCR analysis found that the overexpression of lncRNA ENSMUST_147219 increased the expression of lncRNA ENSMUST_147219 under basic and I/R treatment (Fig. 3A). The FCM results showed that the overexpression of lncRNA ENSMUST_147219 enhanced the I/R-induced apoptosis in BUMPT cells (Fig. 3B, C). The immunoblot analysis verified that the I/R-induced accumulation of cleaved caspase-3 was markedly enhanced by the overexpression of lncRNA ENSMUST_147219 (Fig. 3D, E). These data verified the findings of previous knockdown experiments and confirmed that lncRNA ENSMUST_147219 plays a proapoptotic role during ischemic injury.

### MiR-221-5p is a direct target of lncRNA ENSMUST_147219

**Fig. 4:**
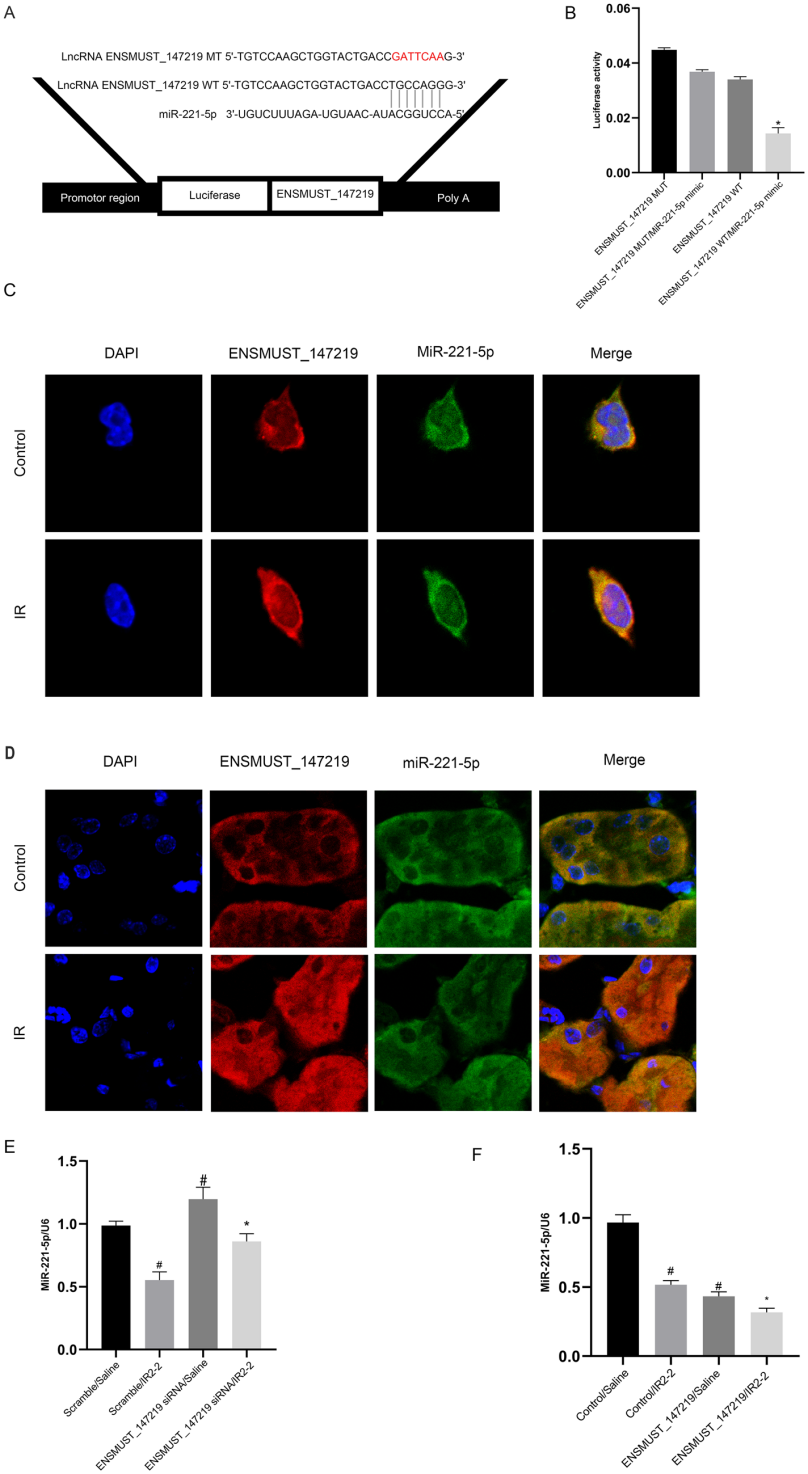
LncRNA ENSMUST_147219 directly binds to miR-221-5p. **A** Sequence alignment analysis indicated that lncRNA ENSMUST_147219 contained the complementary region of miR-221-5p. **B** Detection of luciferase activities after co-transfection of miR-221-5p with ENSMUST_147219-WT or ENSMUST_147219-MUT. **C** and **D** Intracellular colocalization of ENSMUST_147219 and miR-221-5p in BUMPT cells and mice kidney samples under basic and I/R treatment. **E** and **F** RT-qPCR analysis of miR-221-5p expression. ^#^*P* < 0.05, scramble with I/R2-2 group, lncRNA ENSMUST_147219 siRNA or plasmids with saline group versus scramble with saline group; *P < 0.05, lncRNA ENSMUST_147219 siRNA or plasmids with I/R2-2 group versus scramble with I/R2-2 group or lncRNA ENSMUST_147219 WT plus miR-221-5p mimics group versus other groups

Most studies have reported that lncRNAs serve as ceRNAs to sponge miRNAs [29–31]. The RegRNA 2.0 software was used to predict target miRNAs of lncRNA ENSMUST_147219. The prediction results showed that lncRNA ENSMUST_147219 contained the complementary sequence of miR-221-5p (Fig. 4A). The luciferase reporter assays indicated that the luciferase activity of lncRNA ENSMUST_147219-wild-type (WT) but not that of ENSMUST_147219-mutant (MUT) was markedly inhibited by the miR-221-5p mimics (Fig. 4B). The colocalization analysis of lncRNA ENSMUST_147219 and miR-221-5p showed that they localized to the cytosolic compartment of BUMPT cells and renal tubular cells of mice kidneys, and interacted with each other under basic and I/R treatment (Fig. 4C, D). Finally, we found that the expression of miR-221-5p was reversed by lncRNA ENSMUST_147219 knockdown under basic and I/R treatment; in contrast, this reverse effect was enhanced by the overexpression of lncRNA ENSMUST_147219 (Fig. 4E, F). Collectively, these data suggest that lncRNA ENSMUST _147219 acts as ceRNA to sponge the miR-221-5p.

### MiR-221-5p mimics attenuate I/R-induced renal cell apoptosis

**Fig. 5:**
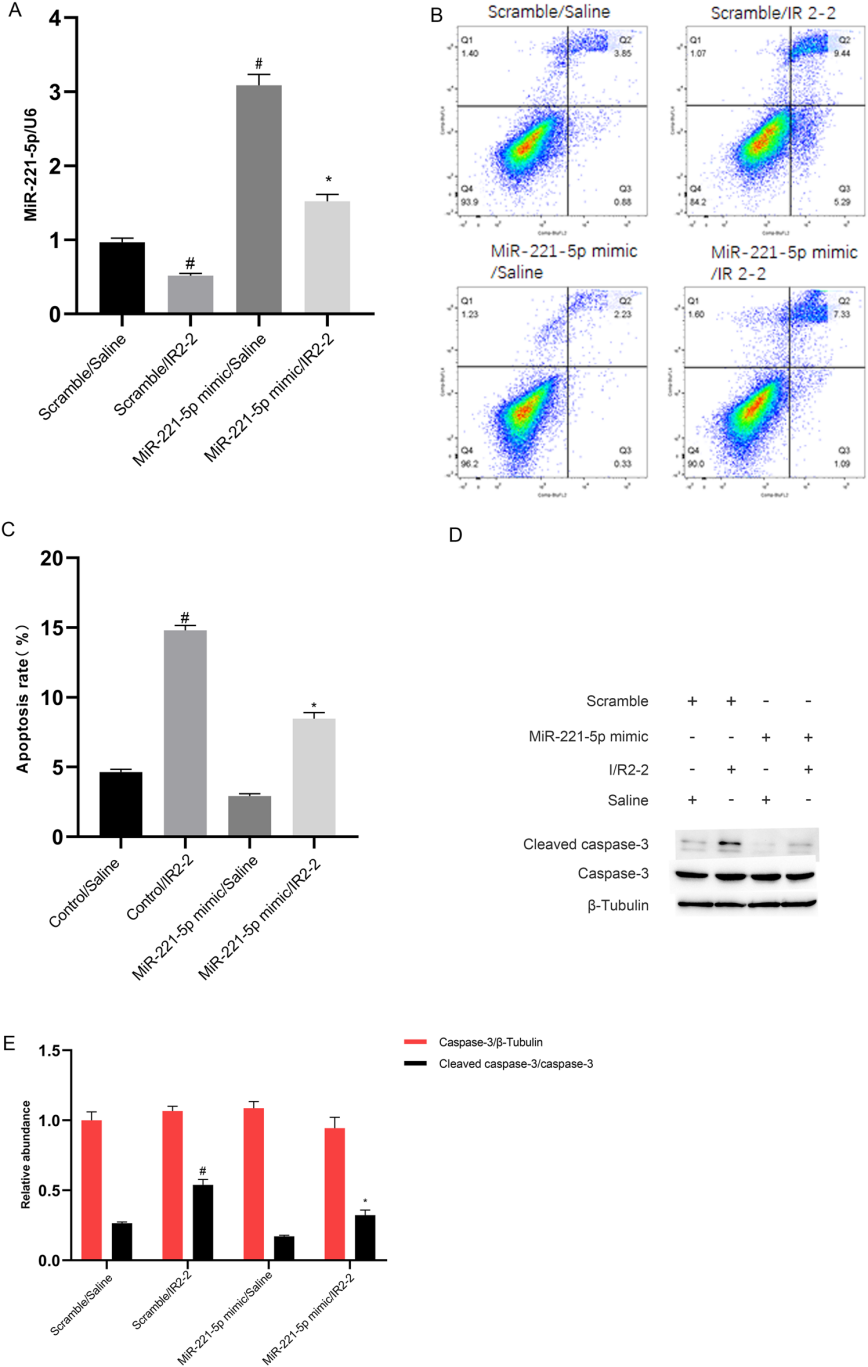
I/R-induced BUMPT cell apoptosis was attenuated by miR-221-5p mimics. BUMPT cells were transfected with 100 nM miR-221-5p mimics or scramble and then subjected to I(2 h)/R(2 h). **A** RT-qPCR analysis of miR-221-5p expression. **B** and **C** The FCM analysis of BUMPT cells apoptosis. **D** Immunoblot analysis of cleaved caspase-3, caspase-3, and β-tubulin. **E** Densitometric analysis of immunoblot bands. Data are expressed as mean ± SD (n = 6). ^#^*P* < 0.05, scramble with I/R2-2 group or miR-221-5p mimic with saline group versus scramble with saline group; *P < 0.05, miR-221-5p mimics with I/R2-2 group versus scramble with I/R2-2 group

Previous studies have shown that miR-221-5p promotes several tumor cells [32, 33]. Here, the RT-qPCR analysis indicated that the miR-221-5p mimics increased the expression of miR-221-5p under basic and I/R treatment (Fig. 5A). The FCM analysis demonstrated that I/R-induced renal cell apoptosis in BUMPT cells was significantly reduced by the miR-221-5p mimics (Fig. 5B, C). The immunoblot results showed that I/R-induced increase in cleaved caspase-3 was markedly inhibited by the miR-221-5p mimics (Fig. 5D, E). Hence, these data indicate that miR-221-5p has an antiapoptotic role during ischemic injury.

### IRF6 is a direct miR-221-5p target gene and mediates I/R-induced renal cell apoptosis

**Fig. 6:**
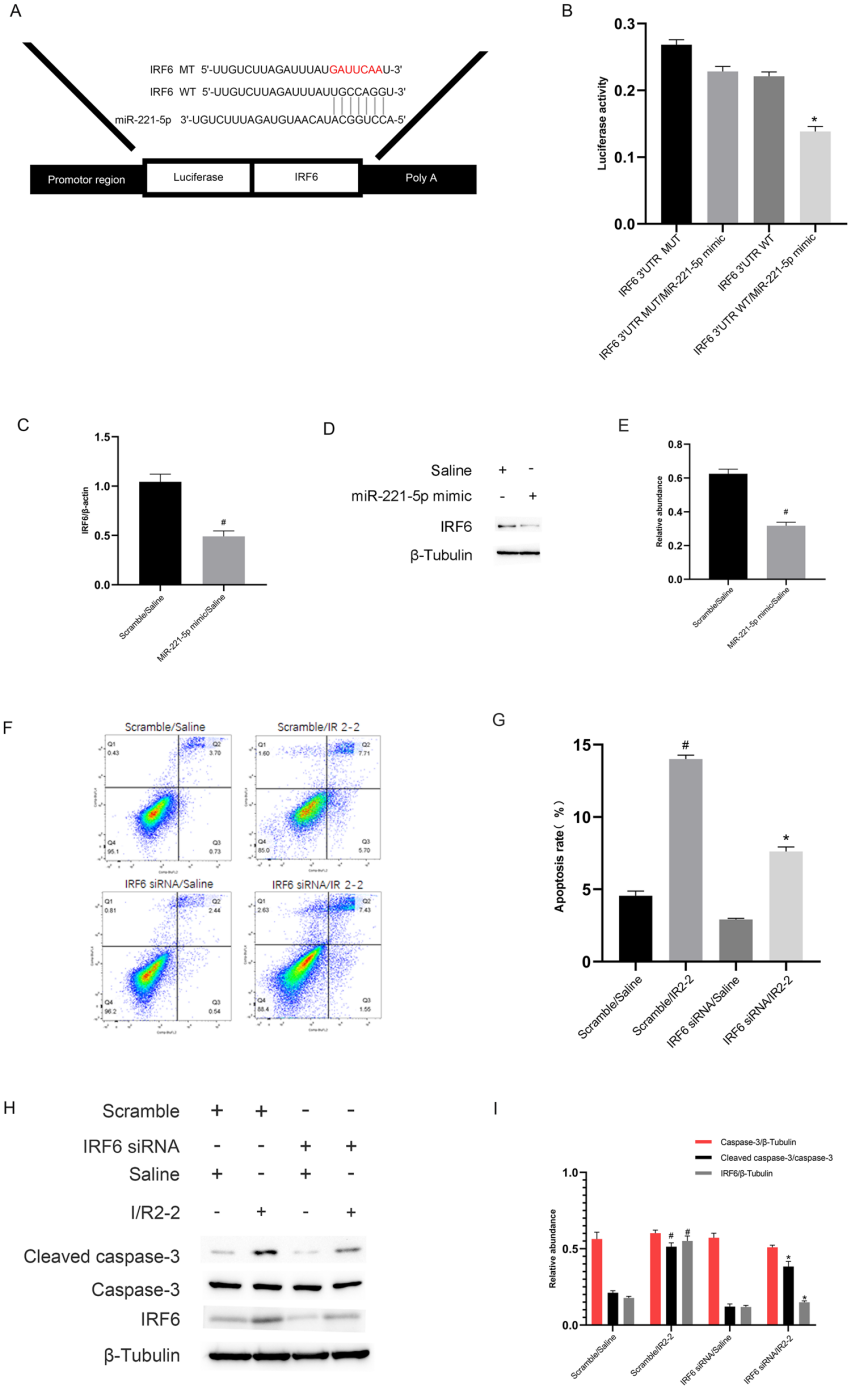
IRF6 is a target gene of miR-221-5p. BUMPT cells were transfected with 100 nM miR-221-5p mimics or IRF6 siRNA or scramble and then subjected to I(2 h)/R(2 h). **A** MiR-221-5p contains the complementary binding sites of IRF6. **B** Measurement of luciferase activities after co-transfection of miR-221-5p mimic or miR-negative control (NC) with the 3′ UTR luciferase reporter vector of WT- or MUT-IRF6. **C** The qRT-PCR of IRF6 and β-actin. **D** and **E** The immunoblot blot analysis of IRF6 and β-tubulin. **F** and **G** The FCM analysis of BUMPT cells apoptosis. **H** Immunoblot analysis of cleaved caspase-3, caspase-3, IRF6, and β-tubulin. **I** Densitometric analysis of immunoblot bands. Data are expressed as mean ± SD (n = 6). ^#^*P* < 0.05, miR-221-5p mimic with saline group or scramble with IR2-2 group versus scramble with the saline group; *P < 0.05, IRF6 siRNA with IR2-2 versus scramble with IR2-2 group or IRF6 3′ UTR WT plus miR-221-5p mimics group versus other groups

Previous studies have reported that IRF6 mediates renal clear cell carcinoma cell apoptosis [34]. The prediction from the miRBase database indicated that miR-221-5p contained the complementary sequence of IRF6 (Fig. 6A). The reporter assays showed that the luciferase activity of IRF6-WT—but not that of IRF6-MUT—was inhibited by the miR-221-5p mimic (Fig. 6B). The results of RT-qPCR and immunoblot showed that the levels of mRNA and protein of IRF6 were suppressed by the miR-221-5p mimics (Fig. 6C–E). In contrast, we found that the expression of IRF6 was reversed by the miR-221-5p inhibitors (Fig. S2G–I). The FCM results verified that IRF6 siRNA notably suppressed I/R-induced renal cell apoptosis in BUMPT cells (Fig. 6F, G). The immunoblot results demonstrated that I/R-induced increase in cleaved caspase-3 was inhibited by the IRF6 siRNA (Fig. 6H, I). Altogether, the data support IRF6 as a direct target of miR-221-5p.

### MiR-221-5p mediates the proapoptotic function of lncRNA ENSMUST_147219

**Fig. 7:**
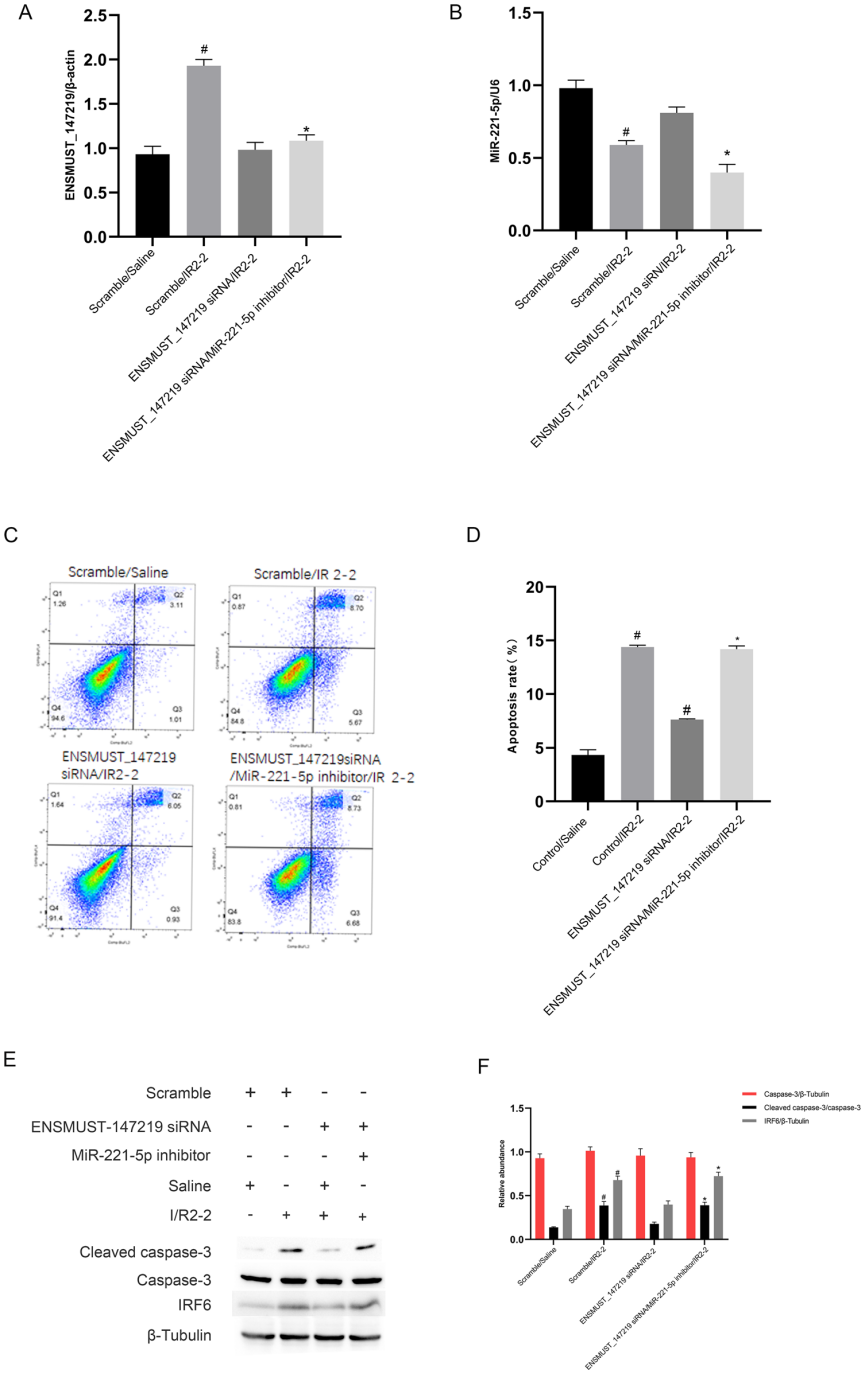
Knockdown of lncRNA ENSMUST_147219 reduced I/R-induced BUMPT cell apoptosis, which was reversed by the miR-221-5p inhibitor. BUMPT cells were co-transfected with ENSMUST_147219 siRNA (100 nM) and anti-miR-221-5p or scramble and then subjected to I(2 h)/R(2 h). **A** RT-qPCR analysis of the expression levels of ENSMUST_147219. **B** RT-qPCR analysis of the expression levels of miR-221-5p. **C** and **D** The FCM analysis of BUMPT cells apoptosis. **E** Immunoblot analysis of cleaved caspase-3 and caspase-3. **F** Densitometric analysis of immunoblot bands. Data are expressed as mean ± SD (n = 6). ^#^*P* < 0.05, scramble or lncRNA ENSMUST_147219 siRNA with I/R2-2 group versus scramble group; *P < 0.05, lncRNA ENSMUST_147219 siRNA plus anti-miR-221-5p with I/R2-2 group versus lncRNA ENSMUST_147219 siRNA with I/R2-2 group

We explored whether the proapoptotic function of lncRNA ENSMUST_147219 was mediated by the miR-221-5p in ischemic injury. The RT-qPCR analysis indicated that lncRNA ENSMUST_147219 siRNA and miR-221-5p inhibitor worked well under basic and I/R conditions (Fig. 7A, B). The FCM analysis showed that the miR-221-5p inhibitor reversed the antiapoptotic effect of lncRNA ENSMUST_147219 siRNA on I/R-induced renal cell apoptosis (Fig. 7C, D), which was further confirmed by the immunoblot analysis of cleaved caspase-3 and IRF6 (Fig. 7E, F). The data verified that lncRNA ENSMUST_147219 promotes the I/R-induced renal cell apoptosis by regulating the miR-221-5p/IRF6 axis.

### Knockdown of lncRNA ENSMUST_147219 attenuates ischemic mice AKI by targeting the miR-221-5p/IRF6 pathway

**Fig. 8:**
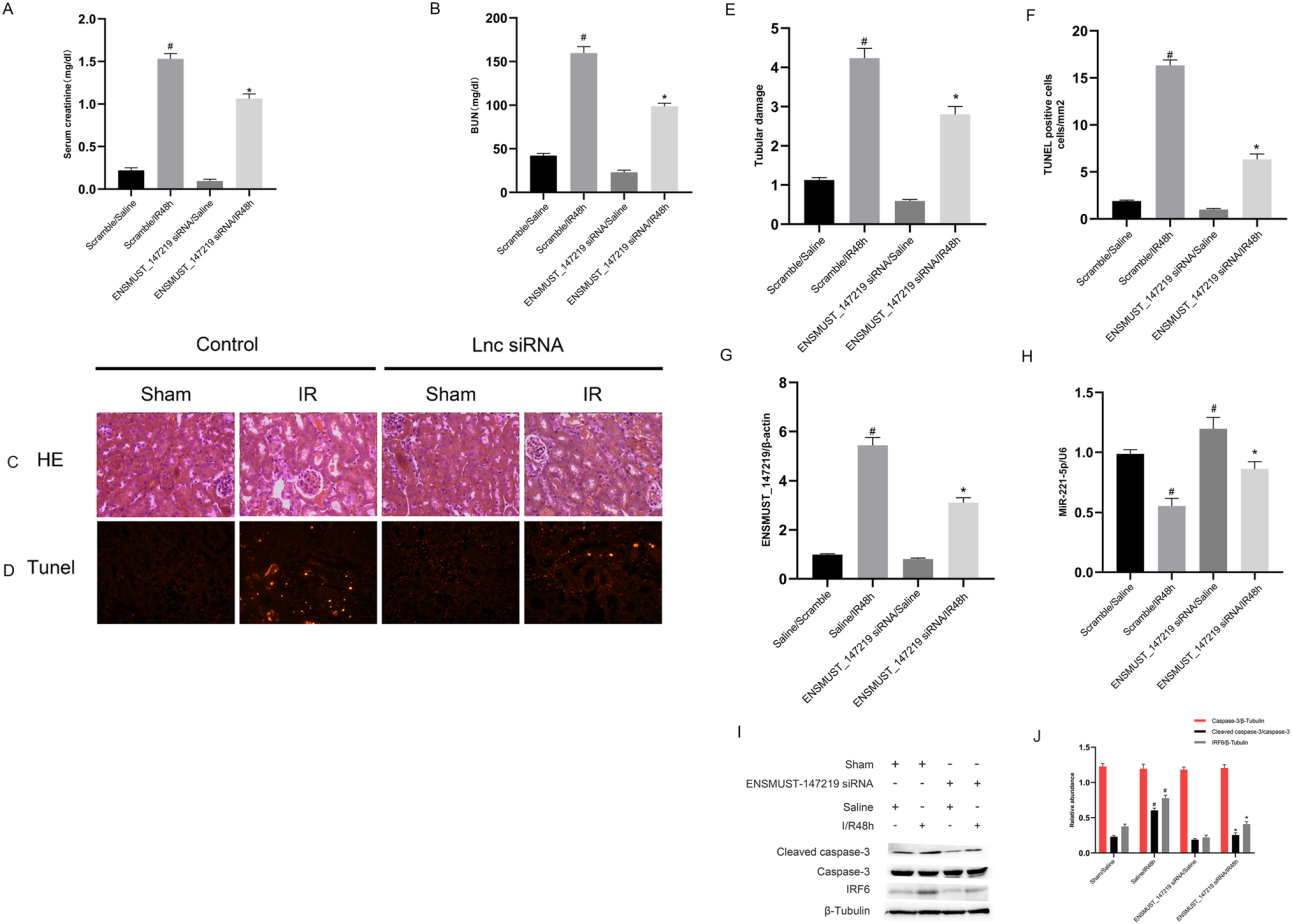
LncRNA ENSMUST_147219 siRNA attenuated I/R-induced AKI in male C57BL/6 mice by targeting the miR-221-5p/IRF6 axis. C57BL/6J mice were preinjected with lncRNA ENSMUST_147219 siRNA or control siRNA via the tail vein for 12 h before ischemic injury, and the blood samples and kidney samples were collected at 48 h after reperfusion. Blood samples were collected for the measurement of serum creatinine (**A**) and serum nitrogen (BUN) (**B**) concentration. The sections of kidney were stained with hematoxylin and eosin (H&E) (**C**) and TUNEL (**D**). **E** Tubular damage scores of the kidney. **F** Counting of TUNEL-positive cells. **G** and **H** RT-qPCR analysis of the expression levels of ENSMUST_147219 and miR-221-5p. **I** Immunoblot analysis of cleaved caspase-3, caspase-3, IRF6, and β-tubulin. **J** Densitometric analysis of immunoblot bands. Data are expressed as mean ± SD (n = 6). ^#^*P* < 0.05, scramble with IR48h group or lncRNA ENSMUST_147219 siRNA group versus saline group; *P < 0.05, lncRNA ENSMUST_147219 siRNA with IR48h group versus control with IR48h group. Original magnification, × 200

To further clarify the role of lncRNA ENSMUST_147219 in ischemic AKI, lncRNA ENSMUST_147219 siRNA was used to suppress lncRNA ENSMUST_147219. The knockdown of lncRNA ENSMUST_147219 noticeably reduced the I/R-induced increase in creatinine and BUN levels (Fig. 8A, B). H&E staining showed that the knockdown of lncRNA ENSMUST_147219 attenuated the I/R-induced tubular damage of mice kidneys (Fig. 8C), which was verified by the tubular damage scores (Fig. 8E). The TUNEL staining showed that the I/R-induced renal cell apoptosis was attenuated by the lncRNA ENSMUST_147219 knockdown (Fig. 8D and F). The RT-qPCR results demonstrated that lncRNA ENSMUST_147219 was suppressed by lncRNA ENSMUST_147219 siRNA (Fig. 8G), while the expression of miR-221-5p was reversed by lncRNA ENSMUST_147219 siRNA under basic and I/R treatment (Fig. 8H). The immunoblot results demonstrated that lncRNA ENSMUST_147219 siRNA markedly suppressed the expression of cleaved caspase-3 and IRF6 (Fig. 8I and J). The data confirmed that lncRNA ENSMUST_147219 mediates the progression of ischemic AKI.

## Discussion

Previous studies have reported that lncRNAs are involved in the progression of ischemic AKI [35, 36]. In the present study, we found that lncRNA ENSMUST_147219 mediated I/R-induced renal cell apoptosis. Mechanistically, lncRNA ENSMUST_147219 sponged the miR-221-5p and then upregulated the expression of IRF6. Finally, the knockdown of lncRNA ENSMUST_147219 ameliorated ischemic AKI via regulating the miR-221-5p/IRF6 axis. Collectively, our findings suggest that lncRNA ENSMUST_147219 promotes the progression of ischemic AKI.

Several studies have reported that lncRNAs regulate the progression of ischemic AKI. One study found that I/R-induced renal cell apoptosis was suppressed by TUG [16]. Other studies showed that I/R-induced renal cell apoptosis was mediated by lncRNA MEG3, lnc NEAT1, lnc GAS5, and lncRNA H19 [17–20]. In the present study, we revealed that lncRNA ENSMUST_147219 also mediated I/R-induced renal cell apoptosis (Fig. 1). Specifically, I/R-induced renal cell apoptosis and the activation of caspase-3 were attenuated by the knockdown of lncRNA ENSMUST_147219 (Fig. 2); however, this effect was enhanced by the overexpression of lncRNA ENSMUST_147219 (Fig. 3). In addition, the knockdown of ENSMUST_147219 ameliorated renal cell apoptosis in I/R-induced AKI (Fig. 8). Taken together, we demonstrated that lncRNA ENSMUST_147219 is an inducer of apoptosis in ischemic AKI.

LncRNAs serve as ceRNA to sponge miRNAs to regulate associated gene expression [37]. In the present study, we demonstrated that miR-221-5p was a target of lncRNA ENSMUST_147219, which was supported by the following levels of evidence: (1) the prediction and dual-luciferase reporter assays showed that lncRNA ENSMUST_147219 bound to miR-221-5p (Fig. 4A and B); (2) the RNA-FISH colocalization of lncRNA ENSMUST_147219 and miR-221-5p supported the findings of the dual-luciferase reporter assays (Fig. 4C and D); (3) the RT-qPCR results showed that lncRNA ENSMUST_147219 negatively regulated the expression of miR-221-5p (Fig. 4E and F). Collectively, the data supported that lncRNA ENSMUST_147219 sponged the miR-221-5p.

Several studies have shown that miR-221-5p is an apoptosis suppressor [38, 39]. In the present study, we also found that miR-221-5p suppressed I/R-induced renal cell apoptosis (Fig. 5). Previous studies have shown that IRF6 is an apoptosis inducer [40, 41]. Our dual-luciferase reporter assays revealed that IRF6 was a target of miR-221-5p (Fig. 6A and B). We further found that the miR-221-5p mimics inhibited the mRNA and protein expression levels of IRF6 (Fig. 6C and D). A previous study indicated that IRF6 mediated renal clear cell carcinoma cell apoptosis [34]. Consistently, we found that IRF6 mediated renal cell apoptosis (Fig. 6E and H). Interestingly, we further confirmed that miR-221-5p mediated the proapoptotic function of lncRNA ENSMUST_147219 (Fig. 7). In addition, the knockdown of lncRNA ENSMUST_147219 attenuated ischemic mice AKI by regulating the miR-221-5p/IRF6 pathway. Collectively, these data suggest that the lncRNA ENSMUST_147219/miR-221-5p/IRF6 axis mediates I/R-induced renal cell apoptosis.

In summary, lncRNA ENSMUST_147219 was found to be an apoptosis inducer during ischemic injury. Mechanistically, lncRNA ENSMUST_147219 sponged miR-221-5p and then increased the expression of IRF6. Finally, the suppression of lncRNA ENSMUST_147219 attenuated ischemic AKI by targeting the miR-221-5p/IRF6 axis. Collectively, our data suggested that lncRNA ENSMUST_147219 may be a novel therapeutic target for ischemic AKI.

## Supplementary Information

Below is the link to the electronic supplementary material.

Supplementary file1 (DOCX 1722 KB)

## Data Availability

The data that support the findings of this study are available from the corresponding author upon reasonable request.

## Abbreviations

BUMPT: Boston University mouse proximal tubule (BUMPT cells)
AKI: Acute kidney injury
lncRNA: Long non-coding RNA
CKD: Chronic kidney disease
FCM: Flow cytometry
I/R: Ischemia/reperfusion
ESRD: End-stage renal disease
