# Supplementary material for: LncRNA ENSMUST_147219 mediates the progression of ischemic acute kidney injury by targeting the miR-221-5p/IRF6 axis
Source: Apoptosis. 2022 May 26;27(7-8):531–44. doi: 10.1007/s10495-022-01730-3 (PMC9308590; doi:10.1007/s10495-022-01730-3)
Supplement: Supplementary file 1 — Supplementary file1 (DOCX 1722 KB) [file 10495_2022_1730_MOESM1_ESM.docx]

**LncRNA ENSMUST_147219 mediates the progression of ischemic acute kidney injury by targeting the miR-221-5p/IRF6 axis**

Jing Liu, Xiaozhou Li, Jurong Yang, Dongshan Zhang


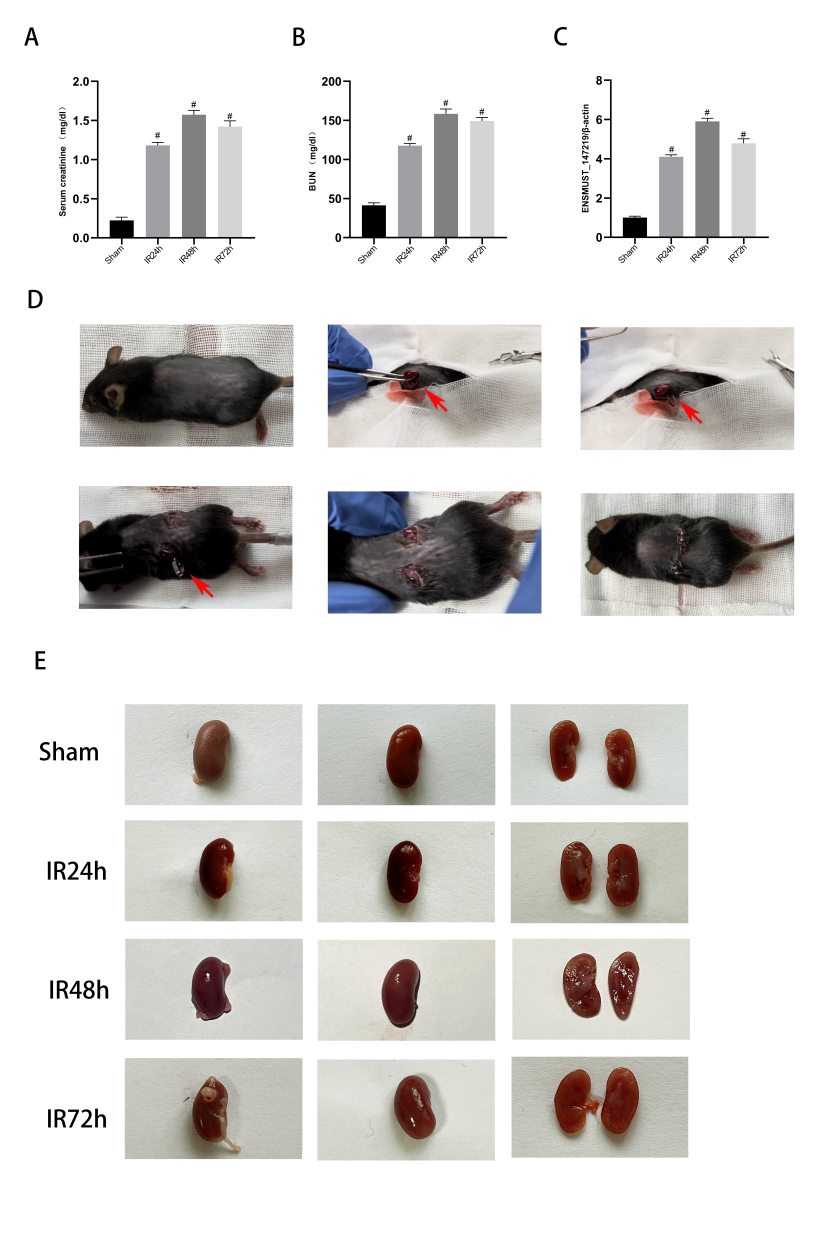


**Supplementary Figure 1:** **The lncRNA ENSMUST_147219 was induced by I/R in vivo**

C57BL/6J mice were subjected to I(30min)/R(24h,48h,72h) treatment. Blood samples were collected for the measurement of serum creatinine (A) and serum nitrogen (BUN) (B) concentrations at 24 h, 48 h and 72h. (C) RT-qPCR analysis of the expression levels of lncRNA ENSMUST_147219. (D) Construction of mouse ischemia-reperfusion model.First, mouse anesthesia and skin preparation.Next, mouse bilateral dorsal incision and exposed kidneys and the bilateral renal arteries of mice were clamped for 30 min at a 37°C heated operation table.And then, remove arterial clips and reperfusion.Finally, clip the wound with skin clips. (E)The morphology of kidney containing envelope, unencapsulated kidney and kidney with exposed cortex and medulla at I(30min)/R24(h)，R48(h)，R72(h).Data are expressed as mean ± SD (n = 6). #*P < 0.05*, versus sham group.


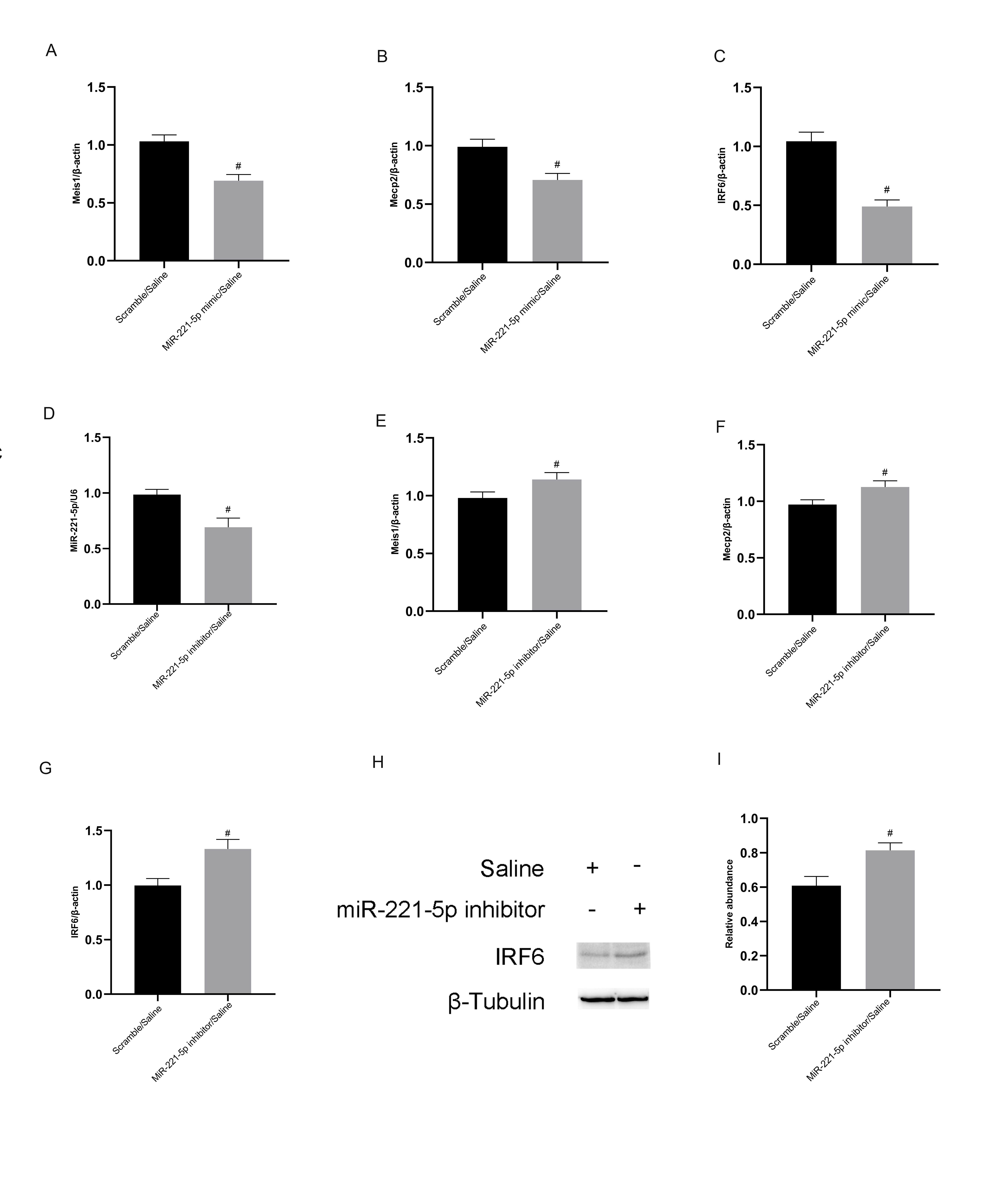


**Supplementary Figure 2: IRF6 is a direct miR-221-5p target gene.** BUMPT cells were transfected with 100 nM miR-221-5p mimic or inhibitor or scramble for 24h. (A) RT-qPCR analysis the expression of Meis1 and β-actin.(B) RT-qPCR analysis the expression of Mecp2 and β-actin.(C) RT-qPCR analysis the expression of IRF6 and β-actin.(D) RT-qPCR analysis the expression of miR-221-5p and U6.(E) RT-qPCR analysis the expression of Meis1 and β-actin. (F)RT-qPCR analysis the expression of Mecp2 and β-actin.(G) RT-qPCR analysis the expression of IRF6 and β-actin.(H) The immunoblot blot analysis of IRF6 and β-tubulin.(I) Densitometric analysis of immunoblot bands.Data are expressed as mean ± SD (n = 6). #*p < 0.05*, miR-221-5p mimic or inhibitor with saline group versus scramble with saline group.
